# Supplementary material for: Correlating in vitro performance with physico-chemical characteristics of nanofibrous scaffolds for skin tissue engineering using supervised machine learning algorithms
Source: R Soc Open Sci. 2020 Dec 23;7(12):201293. doi: 10.1098/rsos.201293 (PMC7813265; doi:10.1098/rsos.201293)
Supplement: Data tables and histology [file rsos201293supp1.pdf]

Royal Society Open Science

Electronic supplementary material

Correlating *in vitro* performance with physico-chemical characteristics of nanofibrous scaffolds for skin tissue engineering using supervised machine learning algorithms

Lakshmi Y. Sujeeun[1,2], Nowsheen Goonoo[1], Honita Ramphul[1], Itisha Chummun[1], Fanny Gimié[3], Shakuntala Baichoo[2], Archana Bhaw-Luximon[1]

[1]*Biomaterials, Drug Delivery and Nanotechnology Unit, Centre for Biomedical and Biomaterials Research (CBBR), University of Mauritius, 80837 Réduit, Mauritius*

[2]*Department of Digital Technologies, Faculty of Information, Communication and Digital Technologies, University of Mauritius, 80837 Réduit, Mauritius*

[3]*Animalerie, Plateforme de recherche CYROI, 2 rue Maxime Rivière, 97490 Sainte Clotilde, Ile de La Réunion, France*

Author for correspondence:
Archana Bhaw-Luximon, Ph.D.
Biomaterials, Drug Delivery and Nanotechnology Unit, Centre for Biomedical and Biomaterials Research (CBBR), University of Mauritius, 80837 Réduit, Mauritius
Tel: (230) 464-3781; Email: abluximon@gmail.com, a.luximon@uom.ac.mu

Table S1 Scaffold data representing the initial dataset of 182 observations, four features (fibre diameter, pore diameter, water contact angle, Young's modulus) and the target variable (number of cells) used in this study.

family_ID	family_name	family_content	triplicates	nb_cells	fibre_diameter	pore_diameter	contact_angle	youngs_modulus
fam_1	PHB/KCG	100/0	t1	53876	2.17	3.4	125.7	545.11
fam_1	PHB/KCG	100/0	t2	54226	0.41	0.72	124.8	476.7
fam_1	PHB/KCG	100/0	t3	60103	1.19	1.75	126.8	532.8
fam_1	PHB/KCG	90/10	t1	46319	1.75	3.73	122	300.3
fam_1	PHB/KCG	90/10	t2	41142	0.13	0.34	118.2	311.4
fam_1	PHB/KCG	90/10	t3	61083	0.62	1.06	119.5	204.3
fam_1	PHB/KCG	80/20	t1	38763	1.5	1.95	108.6	185
fam_1	PHB/KCG	80/20	t2	35614	0.23	0.46	106	123.5
fam_1	PHB/KCG	80/20	t3	32606	0.62	0.89	106.8	174.3
fam_1	PHB/KCG	70/30	t1	45890	1.39	2.91	107.2	79.5
fam_1	PHB/KCG	70/30	t2	42961	0.11	0.42	97.6	91.2
fam_1	PHB/KCG	70/30	t3	25399	0.61	1.02	102.3	101.2
fam_2	PHBV/KCG	100/0	t1	74377	0.63	1.44	121	247.3
fam_2	PHBV/KCG	100/0	t2	76686	0.23	0.28	110.2	182.3
fam_2	PHBV/KCG	100/0	t3	69199	0.42	0.75	107	162
fam_2	PHBV/KCG	90/10	t1	72558	1.53	2.47	75.5	144.1
fam_2	PHBV/KCG	90/10	t2	67100	0.29	0.44	88.3	162.5
fam_2	PHBV/KCG	90/10	t3	39742	0.64	0.9	94.1	173.4
fam_2	PHBV/KCG	80/20	t1	57375	1.35	1.65	62	103.9
fam_2	PHBV/KCG	80/20	t2	44640	0.27	0.23	67.8	136.8
fam_2	PHBV/KCG	80/20	t3	33165	0.64	0.85	63	94.5
fam_2	PHBV/KCG	70/30	t1	42611	1.11	2.7	56.7	103.6
fam_2	PHBV/KCG	70/30	t2	58284	0.22	0.37	65	105.6
fam_2	PHBV/KCG	70/30	t3	37433	0.52	0.85	59.8	116.2
fam_3	PDX/FUC	100/0	t1	33235	0.57	359.9	32.1	72.8
fam_3	PDX/FUC	100/0	t2	38693	0.11	97.51	32.1	66.8
fam_3	PDX/FUC	100/0	t3	25189	0.33	195.01	32.1	81.9
fam_3	PDX/FUC	90/10	t1	3918	0.43	402.66	32.1	64.89
fam_3	PDX/FUC	90/10	t2	6297	0.08	108.99	32.1	64.6
fam_3	PDX/FUC	90/10	t3	12315	0.17	180.24	32.1	79.3
fam_3	PDX/FUC	80/20	t1	16723	0.33	589.59	32.1	42.6
fam_3	PDX/FUC	80/20	t2	15393	0.1	81.76	32.1	36.6
fam_3	PDX/FUC	80/20	t3	8956	0.2	209.37	32.1	35.2
fam_3	PDX/FUC	70/30	t1	13224	0.37	529.93	32.1	47.1
fam_3	PDX/FUC	70/30	t2	25399	0.14	63.25	32.1	33.3
fam_3	PDX/FUC	70/30	t3	24839	0.24	223.79	32.1	24.7

family_ID	family_name	family_content	triplicates	nb_cells	fibre_diameter	pore_diameter	contact_angle	youngs_modulus
fam_4	PDX/KCG	100/0	t1	23439	1.66	3.33	32.1	72.8
fam_4	PDX/KCG	100/0	t2	25189	0.62	0.88	32.1	66.8
fam_4	PDX/KCG	100/0	t3	21550	1.14	2.1	32.1	81.9
fam_4	PDX/KCG	90/10	t1	23650	1.85	2.8	32.1	64.7
fam_4	PDX/KCG	90/10	t2	27708	0.65	0.62	32.1	77.3
fam_4	PDX/KCG	90/10	t3	14134	1.04	1.45	32.1	74
fam_4	PDX/KCG	80/20	t1	20920	1.22	3.2	32.1	35.4
fam_4	PDX/KCG	80/20	t2	14833	0.38	0.73	32.1	45.8
fam_4	PDX/KCG	80/20	t3	14064	0.88	1.29	32.1	46.7
fam_4	PDX/KCG	70/30	t1	34215	0.97	1.45	32.1	32.1
fam_4	PDX/KCG	70/30	t2	30926	0.13	0.51	32.1	42.9
fam_4	PDX/KCG	70/30	t3	26588	0.5	0.85	32.1	39.7
fam_5	PDX/PHBV	100/0	t1	26798	0.65	1.98	32.1	72.8
fam_5	PDX/PHBV	100/0	t2	22880	0.22	0.56	32.1	66.8
fam_5	PDX/PHBV	100/0	t3	25189	0.44	1.04	32.1	81.9
fam_5	PDX/PHBV	90/10	t1	30017	1.6	3.31	32.1	85.6
fam_5	PDX/PHBV	90/10	t2	23230	0.21	0.51	32.1	92.9
fam_5	PDX/PHBV	90/10	t3	21970	0.92	1.59	32.1	108.4
fam_5	PDX/PHBV	80/20	t1	16513	1.29	2.36	103.2	66.6
fam_5	PDX/PHBV	80/20	t2	28617	0.15	0.53	107.6	71.9
fam_5	PDX/PHBV	80/20	t3	24279	0.66	1.21	104.7	80.3
fam_5	PDX/PHBV	70/30	t1	16163	1.16	2.51	116.7	79.3
fam_5	PDX/PHBV	70/30	t2	35754	0.27	0.27	120.7	109
fam_5	PDX/PHBV	70/30	t3	25819	0.61	1.16	121.3	112.8
fam_6	PDX/Psuc	100/0	t1	40610	1.08	14.66	32.1	46.37
fam_6	PDX/Psuc	100/0	t2	28268	1	4.03	32.1	45.25
fam_6	PDX/Psuc	100/0	t3	38245	1.02	7.98	32.1	63.68
fam_6	PDX/Psuc	90/10	t1	29863	0.69	10.78	32.1	54.79
fam_6	PDX/Psuc	90/10	t2	18612	0.96	2.42	32.1	35.44
fam_6	PDX/Psuc	90/10	t3	52687	0.85	5.51	32.1	38.73
fam_6	PDX/Psuc	80/20	t1	33473	0.81	8.15	32.1	28.77
fam_6	PDX/Psuc	80/20	t2	93423	0.76	2.69	32.1	37.46
fam_6	PDX/Psuc	80/20	t3	45214	0.76	4.44	32.1	28.22
fam_6	PDX/Psuc	70/30	t1	29611	0.65	7.61	32.1	81.93
fam_6	PDX/Psuc	70/30	t2	37084	0.72	1.36	32.1	60.05
fam_6	PDX/Psuc	70/30	t3	57641	0.76	3.45	32.1	107.76
fam_6	PDX/Psuc	60/40	t1	49510	0.74	6.07	32.1	80.1
fam_6	PDX/Psuc	60/40	t2	185110	0.69	1.55	32.1	67.25
fam_6	PDX/Psuc	60/40	t3	68724	0.7	3.29	32.1	27.32

family_ID	family_name	family_content	triplicates	nb_cells	fibre_diameter	pore_diameter	contact_angle	youngs_modulus
fam_6	PDX/Psuc	50/50	t1	26504	0.7	6.18	32.1	32.14
fam_6	PDX/Psuc	50/50	t2	2911	0.63	1.51	32.1	35.22
fam_6	PDX/Psuc	50/50	t3	24979	0.63	2.56	32.1	31.6
fam_7	PLLA/Psuc	100/0	t1	31961	1.09	9.36	140.3	235
fam_7	PLLA/Psuc	100/0	t2	57403	1	2.87	143.6	250
fam_7	PLLA/Psuc	100/0	t3	64861	1.05	4.89	140	220
fam_7	PLLA/Psuc	90/10	t1	64120	0.98	6.64	134.1	119
fam_7	PLLA/Psuc	90/10	t2	37503	0.93	1.63	134.3	104
fam_7	PLLA/Psuc	90/10	t3	58312	0.87	4.22	136.9	104
fam_7	PLLA/Psuc	80/20	t1	55388	0.78	6.58	134.3	118
fam_7	PLLA/Psuc	80/20	t2	42373	0.82	1.92	134.4	110
fam_7	PLLA/Psuc	80/20	t3	70571	0.84	3.16	133.2	115
fam_7	PLLA/Psuc	70/30	t1	57235	0.76	5.13	131.3	90
fam_7	PLLA/Psuc	70/30	t2	18276	0.71	1.16	125.5	88
fam_7	PLLA/Psuc	70/30	t3	79639	0.69	2.45	122.1	79
fam_7	PLLA/Psuc	60/40	t1	52701	0.66	2.81	131.7	96
fam_7	PLLA/Psuc	60/40	t2	37671	0.67	1.21	133.6	85
fam_7	PLLA/Psuc	60/40	t3	57641	0.69	2.01	133.5	91
fam_7	PLLA/Psuc	50/50	t1	55388	0.63	3.98	92.1	97
fam_7	PLLA/Psuc	50/50	t2	37839	0.66	1.04	78.8	132
fam_7	PLLA/Psuc	50/50	t3	31192	0.61	2.05	73.8	69
fam_8	PLLA/CA	100/0	t1	31961	1.26	9.36	142.5	235.34
fam_8	PLLA/CA	100/0	t2	57403	0.66	2.87	140.6	250.16
fam_8	PLLA/CA	100/0	t3	64861	0.96	4.8	139	220.59
fam_8	PLLA/CA	0/100	t1	2995	0.63	1.1	132.8	105.84
fam_8	PLLA/CA	0/100	t2	12147	0.8	0.52	134.1	75.2
fam_8	PLLA/CA	0/100	t3	14987	0.72	0.87	136.4	90.04
fam_8	PLLA/CA	90/10	t1	49258	1.15	2.67	138.7	229.17
fam_8	PLLA/CA	90/10	t2	29779	0.61	1.91	137.5	250.44
fam_8	PLLA/CA	90/10	t3	80646	0.88	2.28	138.9	275.91
fam_8	PLLA/CA	80/20	t1	21718	1.17	3.29	139.9	139.6
fam_8	PLLA/CA	80/20	t2	13994	0.47	2.21	140	191.36
fam_8	PLLA/CA	80/20	t3	20445	0.82	2.7	138.8	188.96
fam_8	PLLA/CA	70/30	t1	8452	0.83	4.63	119.6	55.66
fam_8	PLLA/CA	70/30	t2	21802	0.4	2.59	118.5	62.32
fam_8	PLLA/CA	70/30	t3	20865	0.62	3.32	120.3	4.99
fam_8	PLLA/CA	60/40	t1	21551	0.8	3.18	127.5	41.16
fam_8	PLLA/CA	60/40	t2	17772	0.24	1.75	127.2	23.63
fam_8	PLLA/CA	60/40	t3	28002	0.52	2.42	128.5	58.78

family_ID	family_name	family_content	triplicates	nb_cells	fibre_diameter	pore_diameter	contact_angle	youngs_modulus
fam_8	PLLA/CA	50/50	t1	3498	0.68	2.61	116.5	92.7
fam_8	PLLA/CA	50/50	t2	8704	0.28	1.44	118.8	119.6
fam_8	PLLA/CA	50/50	t3	10453	0.48	1.91	116.6	100.9
fam_9	PLLA/cellulose	0/100	t1	16681	0.37	2.04	25	143.21
fam_9	PLLA/cellulose	0/100	t2	31962	0.17	0.87	25	185.3
fam_9	PLLA/cellulose	0/100	t3	46390	0.27	1.5	25	158.51
fam_9	PLLA/cellulose	90/10	t1	16849	1.16	2.67	97.4	300.74
fam_9	PLLA/cellulose	90/10	t2	33725	0.52	1.91	96.7	233.71
fam_9	PLLA/cellulose	90/10	t3	51511	0.84	2.28	97.5	293.51
fam_9	PLLA/cellulose	80/20	t1	19451	0.94	3.29	25	140.42
fam_9	PLLA/cellulose	80/20	t2	26085	0.46	2.21	25	158.83
fam_9	PLLA/cellulose	80/20	t3	57725	0.7	2.7	25	140.37
fam_9	PLLA/cellulose	70/30	t1	6185	0.8	4.63	25	89.47
fam_9	PLLA/cellulose	70/30	t2	43381	0.41	2.59	25	94.89
fam_9	PLLA/cellulose	70/30	t3	49916	0.61	3.32	25	102.76
fam_9	PLLA/cellulose	60/40	t2	45816	0.34	2.64	25	36.41
fam_9	PLLA/cellulose	60/40	t3	15071	0.53	3.31	25	18.85
fam_9	PLLA/cellulose	50/50	t1	42038	0.34	2.85	25	177.78
fam_9	PLLA/cellulose	50/50	t2	34733	0.19	1.78	25	97.63
fam_9	PLLA/cellulose	50/50	t3	19941	0.27	2.27	25	32.39
fam_10	PDX/CA	100/0	t1	40610	1.08	14.66	32.1	46.37
fam_10	PDX/CA	100/0	t2	28268	0.73	4.03	32.1	45.25
fam_10	PDX/CA	100/0	t3	38245	1.2	7.98	32.1	63.68
fam_10	PDX/CA	90/10	t1	41954	1.33	4.67	32.1	40.54
fam_10	PDX/CA	90/10	t2	48167	0.66	1.76	32.1	47.33
fam_10	PDX/CA	90/10	t3	45718	1	3.12	32.1	49.37
fam_10	PDX/CA	80/20	t1	31458	1.43	3.61	32.1	95.56
fam_10	PDX/CA	80/20	t2	37420	0.48	1.7	32.1	73.06
fam_10	PDX/CA	80/20	t3	38833	0.96	2.49	32.1	104.12
fam_10	PDX/CA	70/30	t1	35824	1	7.03	32.1	118.4
fam_10	PDX/CA	70/30	t2	7865	0.51	2.8	32.1	135.71
fam_10	PDX/CA	70/30	t3	57305	0.76	4.52	32.1	149.37
fam_10	PDX/CA	60/40	t1	17017	0.95	2.24	32.1	167.19
fam_10	PDX/CA	60/40	t2	7949	0.33	1.12	32.1	215.12
fam_10	PDX/CA	60/40	t3	24895	0.64	1.83	32.1	192.97
fam_10	PDX/CA	50/50	t1	34061	0.74	2.73	32.1	74.81
fam_10	PDX/CA	50/50	t2	18612	0.38	1.76	32.1	49.57
fam_10	PDX/CA	50/50	t3	38665	0.56	2.17	32.1	72.76
fam_11	PLLA/CA 1% NS	100/0	t1	48755	1.08	9.36	128.2	149.4

family_ID	family_name	family_content	triplicates	nb_cells	fibre_diameter	pore_diameter	contact_angle	youngs_modulus
fam_11	PLLA/CA 1% NS	100/0	t2	68570	0.91	2.87	129.5	176.44
fam_11	PLLA/CA 1% NS	100/0	t3	57725	1	4.91	128.1	178.96
fam_11	PLLA/CA 1% NS	90/10	t1	22726	0.67	2.78	130.1	126.92
fam_11	PLLA/CA 1% NS	90/10	t3	44458	0.59	2.25	128.4	138.43
fam_11	PLLA/CA 1% NS	70/30	t1	10551	0.51	3.71	129.6	62.57
fam_11	PLLA/CA 1% NS	70/30	t2	23230	0.32	1.89	131.9	60.85
fam_11	PLLA/CA 1% NS	70/30	t3	12217	0.42	2.65	129.6	77.12
fam_11	PLLA/CA 1% NS	50/50	t1	2911	0.44	2.89	123.2	77.25
fam_11	PLLA/CA 1% NS	50/50	t2	20627	0.28	1.9	123.6	95.7
fam_11	PLLA/CA 1% NS	50/50	t3	5332	0.36	2.42	124	94.56
fam_12	PLLA/cellulose 1% NS	90/10	t1	51189	0.7	2.82	103.8	60.01
fam_12	PLLA/cellulose 1% NS	90/10	t2	32886	0.61	1.78	117.6	82.1
fam_12	PLLA/cellulose 1% NS	90/10	t3	40344	0.66	2.22	104.7	85.79
fam_12	PLLA/cellulose 1% NS	70/30	t1	46236	0.47	2.59	25	30.59
fam_12	PLLA/cellulose 1% NS	70/30	t2	20375	0.36	1.72	25	43.49
fam_12	PLLA/cellulose 1% NS	70/30	t3	56885	0.42	2.13	25	71.23
fam_12	PLLA/cellulose 1% NS	50/50	t1	21634	0.3	2.4	25	172.92
fam_12	PLLA/cellulose 1% NS	50/50	t2	43969	0.21	1.36	25	90.41
fam_12	PLLA/cellulose 1% NS	50/50	t3	26574	0.26	1.8	25	142
fam_13	PDX/CA 1% NS	100/0	t1	39267	0.88	1.88	69.8	50.93
fam_13	PDX/CA 1% NS	100/0	t2	48755	0.77	1	32.1	63.58
fam_13	PDX/CA 1% NS	100/0	t3	63434	0.83	1.43	32.1	66.78
fam_13	PDX/CA 1% NS	90/10	t1	74195	1.06	3.41	32.1	66.87
fam_13	PDX/CA 1% NS	90/10	t2	48922	0.85	1.74	32.1	60.92
fam_13	PDX/CA 1% NS	90/10	t3	77960	0.96	2.3	32.1	76.48
fam_13	PDX/CA 1% NS	60/40	t1	28435	0.74	3.24	32.1	150.58
fam_13	PDX/CA 1% NS	60/40	t2	18024	0.48	1.8	32.1	160.57
fam_13	PDX/CA 1% NS	60/40	t3	58648	0.61	2.45	32.1	154.44

Table S2 Nanoscaffolds found in the 95% confidence interval while plotting the actual values from the test dataset against predicted values from the random forest model.

Nanoscaffolds	Polymer blends	Triplicates	Actual/Observed MTT Number of cells after 7 days	Predicted MTT Number of cells	Fibre diameter (μm)	Pore diameter (μm)	Water contact angle (°)	Young's modulus (MPa)
PDX	100	t2	25189	31508	0.62	0.88	32.1	66.8
PDX	100	t1	40610	39525	1.08	14.66	32.1	46.37
PDX/CA	90/10	t2	48167	36829	0.66	1.76	32.1	47.33
PDX/CA	70/30	t3	57305	42575	0.76	4.52	32.1	149.37
PDX/CA 1% NS	90/10	t2	48922	40694	0.85	1.74	32.1	60.92
PDX/KCG	70/30	t1	34215	35622	0.97	1.45	32.1	32.1
PDX/Psuc	80/20	t1	33473	36866	0.81	8.15	32.1	28.77
PDX/Psuc	60/40	t3	68724	40872	0.7	3.29	32.1	27.32
PHB/KCG	80/20	t3	32606	45950	0.62	0.89	106.8	174.3
PHB/KCG	80/20	t2	35614	38030	0.23	0.46	106	123.5
PHB/KCG	90/10	t2	41142	50109	0.13	0.34	118.2	311.4
PHB/KCG	90/10	t3	61083	44880	0.62	1.06	119.5	204.3
PHBV/KCG	80/20	t1	57375	37554	1.35	1.65	62	103.9
PLLA	100	t3	64861	45561	1.05	4.89	140	220
PLLA/cellulose	60/40	t3	15071	32138	0.53	3.31	25	18.85
PLLA/cellulose 1% NS	70/30	t2	20375	31441	0.36	1.72	25	43.49
PLLA/Psuc	50/50	t2	37839	39270	0.66	1.04	78.8	132
PLLA/Psuc	70/30	t3	79639	46285	0.69	2.45	122.1	79

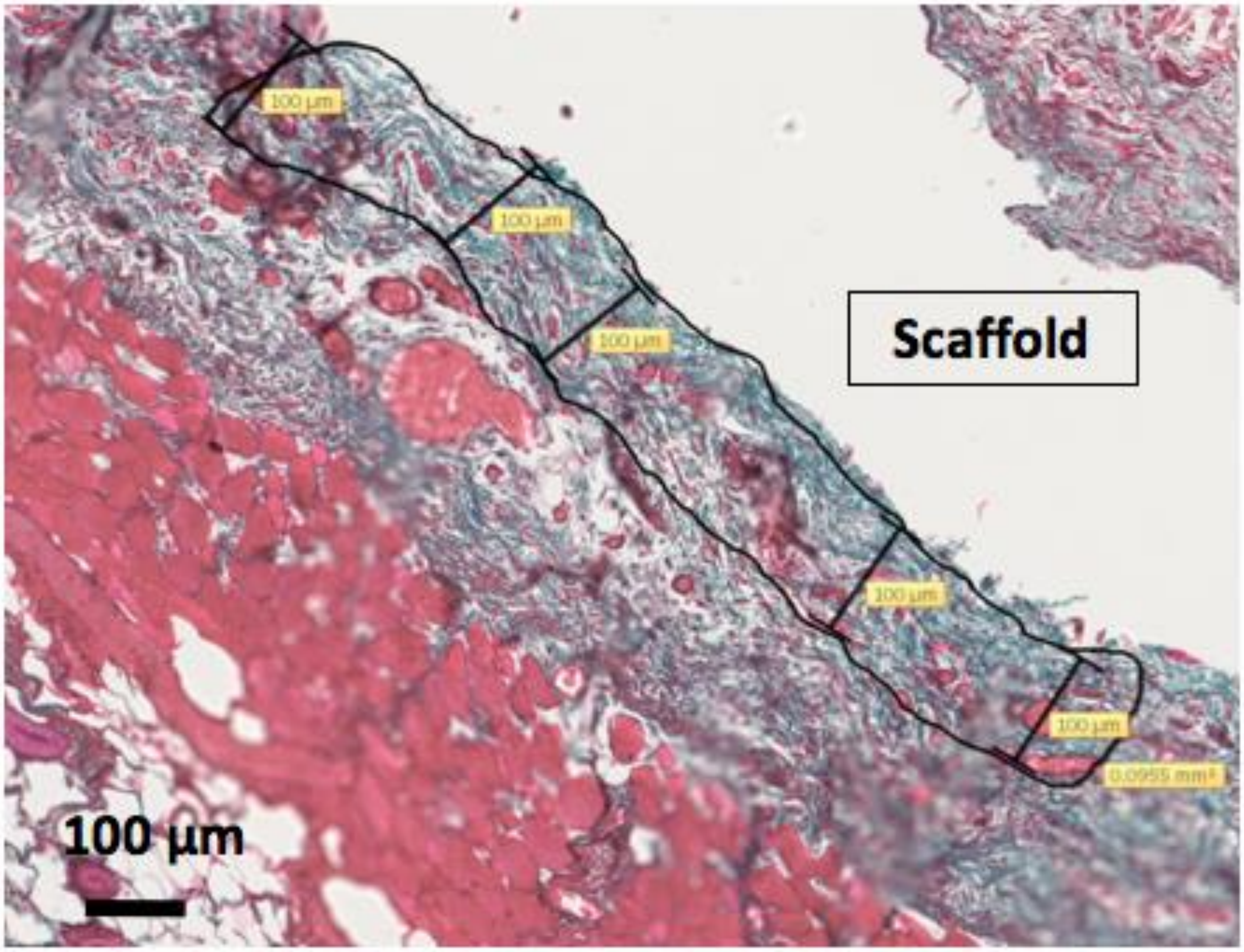

Figure S1 Histology image showing Massons's Trichrome staining of tissue around scaffold. The number of blood vessels within the black designated area was counted and expressed as number of blood vessels/mm^2.
